# The Association between Dietary Purine Intake and Mortality: Evidence from the CHNS Cohort Study

**DOI:** 10.3390/nu14091718

**Published:** 2022-04-21

**Authors:** Miaojia Yan, Yezhou Liu, Lichen Wu, Huimeng Liu, Yutong Wang, Fangyao Chen, Leilei Pei, Yaling Zhao, Lingxia Zeng, Shaonong Dang, Hong Yan, Baibing Mi

**Affiliations:** Department of Epidemiology and Biostatistics, School of Public Health, Xi’an Jiaotong University Health Science Center, No.76, Yanta West Road, Xi’an 710061, China; yanmj01@stu.xjtu.edu.cn (M.Y.); yezhouliu@stu.xjtu.edu.cn (Y.L.); wlc15211028421@stu.xjtu.edu.cn (L.W.); xjtu.lhm@stu.xjtu.edu.cn (H.L.); wangyut@stu.xjtu.edu.cn (Y.W.); chenfy@mail.xjtu.edu.cn (F.C.); peileilei424@163.com (L.P.); zhaoyl666@xjtu.edu.cn (Y.Z.); tjzlx@xjtu.edu.cn (L.Z.); tjdshn@xjtu.edu.cn (S.D.); yanhonge@xjtu.edu.cn (H.Y.)

**Keywords:** purine, mortality, CHNS, Chinese adults, cohort study

## Abstract

Objectives: To investigate the association between dietary purine intake and mortality among Chinese adults. Methods: Based on data from the 2004–2015 China Health and Nutrition Survey (CHNS) and the corresponding edition of China Food Composition, the average purine intake per day (mg/day) from 2004 to 2011 was calculated, and the surveyed population was divided into five groups by quintiles. The outcome event and timepoint of concern were defined as death and time, respectively, as reported by family members, recorded until the 2015 survey. Cox proportional hazards regression was used to estimate the hazard ratios (HRs) with 95% confidence intervals (CIs) for death. The possibly nonlinear relationship between purine intake and mortality was examined with restricted cubic splines. Results: We included 17,755 subjects, and the average purine intake among them was 355.07 ± 145.32 mg/day. Purine intake was inversely associated with mortality (*P_trend_* < 0.001). Compared with the lowest quintiles of purine intake, the highest quintiles (HR = 0.60; 95% CI: 0.46, 0.77) showed a significant association with lower mortality. The negative association with mortality was mainly found in plant-derived purine (*P_trend_* = 0.001) and, weakly, in animal-derived purine (*P_trend_* = 0.052). In addition, a U-shaped relationship between purine intake and mortality was observed in males; however, there was no statistically significant dose–response relationship in females. *Conclusion:* Considering the low-purine-intake levels of the Chinese population, we observed a U-shaped relationship between purine intake and mortality in males, but purine intake may not relate to mortality in females. Future studies should investigate the causal relationship between purine intake and disease burden in China.

## 1. Introduction

As key compounds in cellular signal transduction, purines play important roles in various physiological processes, such as cell proliferation, differentiation, and apoptosis [1]. Hyperuricemia and gout can result from the accumulation of uric acid caused by abnormal purine metabolism. Therefore, to prevent and treat hyperuricemia and gout, it is recommended to eat fewer purine-rich foods [2,3].

The incidence and frequency of gout and hyperuricemia increase globally with economic growth and lifestyle changes. According to statistics on Europeans and Americans, the incidence of gout is 3–6% in males and 1–2% in females [4]. According to a systematic review, the prevalence of gout is 0.4–1.5% in China [5]. In addition, the increased risk of comorbidities and mortality due to hyperuricemia and gout cannot be ignored. A 2007–2008 study showed that 74% of gout patients had hypertension and 10% had a stroke history in the US [6]. Gout may increase the risk of death, especially from cardiovascular disease [7].

In recent years, many studies have shown the link between the consumption of purine-rich foods and these two diseases. Studies have shown that the intake of purine-rich foods such as meat and seafood is associated with elevated blood uric acid levels and an increased risk of gout and hyperuricemia [8,9]. However, purine-rich vegetables did not affect gout or serum uric acid levels [8,10]. A cross-sectional study in China suggested that purine-rich soy products were associated with a lower prevalence of hypouricemia, while animal products and seafood were associated with a higher prevalence of hyperuricemia [11,12].

Nevertheless, most relevant studies have focused on the relationship between dietary purine intake and hyperuricemia and gout, and few have been conducted with a focus on mortality. Since death is the ultimate manifestation of ill health, studying the relationship between purine intake and death can help clarify the role of purines in human health. In addition, the conclusions of these studies may not apply to the Chinese population due to differences in dietary habits, ethnicity, and genetics between China and other countries. Furthermore, most of the aforementioned studies have examined the relationship between purine-rich foods and disease, but quantitative studies on purine intake and health outcomes are lacking.

This study uses data from the China Nutrition and Health Survey (CHNS), a nationally representative cohort study [13], to explore the relationship between quantitative dietary purine intake and mortality in Chinese adults.

## 2. Methods

### 2.1. Study Design and Participants

The CHNS is a nationally representative long-term-follow-up survey project. The content of the survey includes general demographics, lifestyle, health status, diet, etc. Since its establishment in 1989, CHNS has been used to collect 10-panel data in 1989, 1991, 1993, 1997, 2000, 2004, 2006, 2009, 2011, and 2015, and has been used to survey 42,829 participants cumulatively from 12 provinces and three megacities in China (three new cities were added in 2011, and three new provinces were added in 2015). These areas vary in levels of economic development, public resources, geographic location, and citizen health. A multistage random-cluster sampling method was used to obtain research samples in each province or city for representativeness.

**Figure 1:**
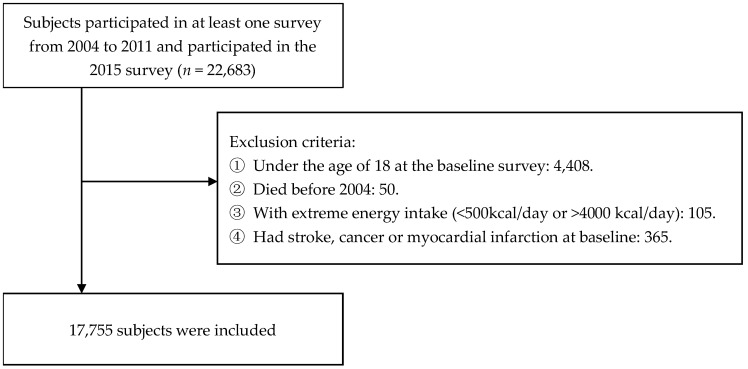
Flow chart of subject exclusion.

Our study included adults who participated in at least one dietary recall during the four waves of CHNS from 2004 to 2011. We used survey data from 2004 to 2011 to measure cumulative average dietary purine intake individually. The data recorded until the 2015 survey were used to determine the survival status and times of the subjects we included. Between 2004 and 2011, a total of 22,683 subjects participated in the survey. Participants under the age of 18 (*n* = 4408) and those who died before 2004 (*n* = 50) were excluded. Due to unreliable dietary intake data, subjects with excessively low or high energy intake (<500 kcal/day or >4000 kcal/day, *n* = 105) were excluded [14,15]. Furthermore, subjects with stroke, cancer, or myocardial infarction at baseline (*n* = 365) were excluded. Finally, 17,755 subjects were included in the analysis. Figure 1 shows the flow chart of the subject-selection process.

### 2.2. Assessment of Purine Intake

Dietary data were collected using three consecutive 24-h recalls at the individual level and a food inventory at the household level [16,17]. The three consecutive days were randomly allocated in a week and were almost equally balanced across the seven days for each sampling unit. Participants reported the consumption of all foods on each of the previous three days individually. The consumption of food at the household level was determined by the weighing method. All foods in the household were weighed and recorded at the beginning and the end of each survey day. Well-trained investigators compared the data between household surveys and individual surveys to determine whether they were consistent. If they were not, investigators revisited. Finally, the type of food (identified by a unique food-ID) and consumption were recorded each day.

The cumulative average daily purine intakes were used to reflect long-term diet. According to the sixth edition of the *Chinese Food Composition Table* [18] and the dietary intake data of the four surveys in 2004, 2006, 2009, and 2011, we first calculated the daily average purine intake in each wave. Next, the cumulative average daily purine intake (mg/day) was calculated as the sum of daily average intake per wave from the first survey to the last survey year divided by the number of waves. For any food lacking a specific measurement value of purine content, the average purine content of its food category was used [19]. We also calculated the cumulative daily purine intake from animal food sources (livestock meat, poultry meat, milk, eggs, and seafood) and plant food sources (cereals, tubers, beans, vegetables, fungi and algae, fruits, nuts, and seeds). Moreover, to explore the relationship between the intake of purine-rich foods and mortality, we calculated the cumulative average daily consumption of purine-rich foods (red meat, poultry, seafood, legumes, purine-rich vegetables, and fungi).

### 2.3. Ascertainment of Deaths

In each survey, family members reported whether the participants had died and recorded the date of death. The death status of each subject was determined by the report of household members. The first report was chosen when reported repeatedly in different waves. Because the survey did not report specific causes of death, we used all-cause mortality as the outcome. Person-years were calculated from the first survey year to the year of death or the end of follow-up (2015).

### 2.4. Assessment of Covariates

Based on the literature and prior knowledge [20,21,22], we considered the following covariates: age at baseline, sex, residence, income, years of education, smoking status, drinking status, physical activity, BMI, energy intake, macronutrient intake, hypertension status, and diabetes status. These general demographic characteristics and lifestyle variables were all collected from adult questionnaires at baseline. Ages at baseline were divided into three groups: 18–40 years old, 40–60 years old, and over 60 years old. Physical activity was converted into metabolic equivalents according to the compendium of physical activities [23]. The energy and macronutrient intake were calculated by the 24-h diet-recall data and the *Chinese Food Composition Table*. BMI was calculated as weight divided by the square of the height (kg/m^2^). The prevalence of hypertension and diabetes was determined by self-reported previous diagnosis by a physician. Uric acid was measured only in the 2009 wave by a blood sample test.

### 2.5. Statistical Analysis

The purine intake was energy-adjusted by the residual method [24]. Subjects were categorized into five groups according to the quintiles of purine intake. Continuous variables are presented as the means ± SDs; categorical variables are presented as percentages. The differences across quintiles were analyzed by the Jonckheere–Terpstra test for continuous variables or the Cochran–Armitage trend test for categorical variables.

We performed multiple imputations of residence (27.02% missing), income (27.02% missing), years of education (1.21% missing), smoking status (1.13% missing), drinking status (1.14% missing), physical activity (17.03% missing), BMI (6.61% missing), hypertension status (1.47% missing), and diabetes status (1.41% missing) using the FCS method [25]. Twenty imputed datasets were produced, and the parameters of each imputed dataset computed by the standard statistical procedure were combined to generate the final results. Multivariate Cox proportional hazard models were used to calculate the hazard ratios (HRs) and 95% CIs for death to evaluate the association between dietary purine intake and mortality. We used Schoenfeld residual plots to assess the proportional hazards assumption. The same methods were used to determine the relationships between animal-derived purine intake, plant-derived purine intake, and five types of purine-rich food and mortality. Model 1 was adjusted for age (18–40 years, 40–60 years, or over 60 years) and sex. Model 2 was additionally adjusted for residence (urban or rural), income (continuous variable, CNY/year), years of education (continuous variable, year), smoking status (yes or no), drinking status (yes or no), physical activity (continuous variable, MET-h/day), BMI (continuous variable, kg/m^2^), and energy intake (continuous variable, kcal/day). Model 3 was further adjusted for disease status, including for hypertension (yes or no) and diabetes (yes or no). The possibly nonlinear relationship between purine intake and mortality was examined with restricted cubic splines (adjusted for age, sex, residence, income, years of education, smoking status, drinking status, physical activity, BMI, energy intake, hypertension status, and diabetes status). Based on Model 3, Model 4 included adjusted protein intake (continuous variable, g/day) to explore whether the association was related to protein. A subgroup analysis was conducted to assess whether the association differed by sex.

All analyses were conducted with SAS 9.4 software. Statistical significance was set at *p* < 0.05 (two-tailed).

## 3. Results

### 3.1. Baseline Characteristics

In total, 17,755 subjects were included in this research, with 9350 (52.66%) females and 8405 (47.34%) males. The average purine intake was 355.07 ± 145.32 mg/day. In total, 758 deaths were documented, with 426 deaths in males and 332 deaths in females. Table 1 shows the baseline characteristics of the participants by quintiles of purine intake. Subjects with higher purine intake were more likely to be male, be younger, dwell in urban settings, have higher education levels, have higher income, currently smoke, currently drink alcohol, and have hypertension. For macronutrient intake, those who consumed more purine consumed fewer carbohydrates, more protein, and less fat. Groups with high purine intake also had higher blood uric acid levels.

### 3.2. Association between Total Purine Intake and Mortality

**Figure 2:**
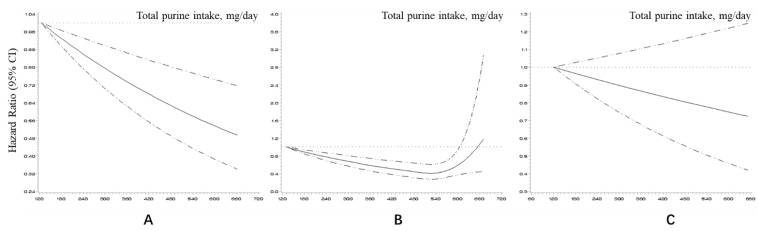
Dose–response relationship between total purine intake and mortality in restricted cubic splines after adjustment (for residence, income, education year, smoking status, drinking status, physical activity, BMI, energy intake, hypertension, and diabetes): (**A**) All participants; (**B**) Male; (**C**) Female.

Table 2 presents the HRs and 95% CIs of mortality according to the quintiles of purine intake. For total purine, as intake increased, HR gradually decreased (*P_trend_* < 0.001). Compared with the lowest quintile, the highest quintile had a 40% decreased risk of death (HR = 0.60; 95% CI: 0.46–0.77). A negative linear dose–response relationship also suggested that higher purine intake was related to lower mortality (Figure 2A). With further adjustment for protein intake, the highest quintile still had a lower HR (0.74, 95% CI: 0.56–0.99), although the trend was not statistically significant (*P_trend_* = 0.144).

### 3.3. Association between Purines from Different Food Sources and Mortality

The HRs and 95% CIs of mortality according to the quintiles of animal-derived purine and plant-derived purine are shown in Table 2, separately. The trend between the purines from animal foods and mortality had a critical *p* value (*P_trend_* = 0.052). The participants in the highest quintile had a lower risk of death than those in the lowest quintile (0.71; 95% CI: 0.55–0.92). Similar to the trend for total purine intake, the trend’s *p* value became insignificant after adjusting for protein intake (*P_trend_* = 0.778).

A higher intake of purines of plant origin was related to lower mortality. It was still significant after it was adjusted for protein intake (*P_trend_* = 0.019). Furthermore, it was observed that the HRs decreased gradually with increasing plant-derived purine intake. Moreover, these HRs were similar to those for the total purine intake.

Table S1 shows the HRs (95% CIs) of mortality according to the quintiles of purine-rich food intake. The intake of these high-purine foods was relatively low. Compared with the lowest quintile, higher-quintile groups had HR values of less than 1. Nevertheless, as the intake increased, the HRs did not show a decreasing trend.

### 3.4. Association between Total Purine Intake and Mortality among Different Genders

The subgroup analysis results are presented in Table 3 (for males) and Table 4 (for females). The association of purine intake with mortality varied between males and females. Similar trends were observed in the males to those in all the participants. The total purine intake and animal-derived purine intake showed protective associations with mortality, but these disappeared after including protein intake in the model. Furthermore, plant-derived purine intake, which is less affected by protein, had a stable, negative association with mortality. However, for females, regardless of the food of origin, purines did not have a significant association with mortality (*P_trend_* > 0.05).

Figure 2 shows the dose–response relationships between the total purine intake and mortality for both sexes (B and C). A U-shaped curve was observed for the males. However, the average purine intake corresponded to the lowest point of the curve, which was approximately 540 mg/day, higher than the highest quintile’s median intake (517.37 mg/day). The dose–response relationship was not statistically significant in the females.

## 4. Discussion

Our research found that higher dietary purine intake may be a protective factor against death, and the dose–response relationship curve suggested that the mortality rate gradually decreased with increasing dietary purine intake. The attenuated association of purine and mortality in all the participants after adjustment for protein intake suggests that this association may have been due to increased protein intake. After a further analysis of purines from different food sources, we found that those who consume more purines derived from plant foods may have lower mortality and are not affected by protein intake. Purines derived from animal food showed a similar association with mortality, but this association was related to protein. In addition, the association between purine intake and mortality differed by sex. In the males, purine intake exhibited a negative association with mortality, and the association of animal-derived purines and mortality was related to protein, while the association of plant-derived purines and mortality was not related to protein. However, purine intake did not show a statistically significant association with mortality in the females, whether it was plant-derived or animal-derived. In the males, the dose–response relationship between purine intake and mortality was a U-shaped curve. In the females, there was a linear dose–response relationship that was not statistically significant.

Surprisingly, this is not consistent with the conclusions of many related studies. Although few studies have focused on the relationship between dietary purine intake and mortality, most existing evidence suggests that foods with high purine content are related to hyperuricemia and gout. These two diseases are risk factors for cardiovascular death [26,27,28]. A cross-sectional study using CHNS data showed that the intake of purine-rich food (not including vegetables) is associated with increased risk of hyperuricemia [12]. Similar findings have been reported in other countries or regions [8,10,29,30,31,32]. We found that the inverse association with mortality was mainly in plant-derived purines, which is consistent with previous research on plant-derived purine-rich foods. We speculate that the following possible mechanisms may explain these findings: First, uric acid is the end product of purine metabolism. Higher uric acid levels are often associated with hyperuricemia and gout and increase the risk of mortality from cardiovascular disease. Previous studies [33] have shown that the consumption of purine-rich animal foods, such as meat and seafood, increases uric acid levels; however, protein intake does not increase uric acid but instead has the effect of lowering uric acid. In our study, after adjusting for protein intake, the corresponding HR value of the animal purine intake increased, and the trend in mortality disappeared. Because purine-rich animal foods are usually rich in protein, the increase in protein intake probably weakened the increase in uric acid and the risk of death. Second, many studies have also proven that there is a J- or U-shaped relationship between uric acid levels and mortality [34,35,36,37]. According to related studies in Japan, the uric acid levels corresponding to the lowest risk of death for men and women are 7 mg/dL and 5 mg/dL, respectively. In our study, the average uric acid level of the highest quintile of purine intake in the Chinese population was 328 μmol/L, close to that at the U-shaped relationship’s low point. The U-shaped dose–response relationship in males also proves this hypothesis: the purine intake at the lowest point of the curve was 540 mg/day, which is close to the median intake of the fifth-quantile group, suggesting that the purine intake of most Chinese adults is below this threshold.

In addition, cooking methods significantly impact the purine content in foods. The high-temperature treatment of foods can reduce purine content. In this study, the effect of cooking methods on purines was not considered due to data limitations. Chinese people usually cook food at high temperatures, so their actual intake of purines may be less than that indicated by our research. Because this study found that the overall consumption of high-purine foods among Chinese people is relatively low, this association may have arisen because the intake level had not yet reached the critical value at which risk is increased. Third, our study used a quantitative method to calculate purine intake, while most previous studies used purine-rich foods for analysis, which may also be a source of the differences between the research conclusions. Perhaps the inverse association of plant-derived purine and mortality can be partially explained by plant-derived purine helping to prevent obesity and reducing insulin resistance [38]. Other protective ingredients in plant foods, such as phytochemicals, may also be responsible for the reduced risk of death owing to their anti-inflammatory and analgesic functions and hypouricemic effects [39,40].

Gender differences in the relationships among purines, diseases, and death are described in many studies [41]. Gender is a factor influencing hyperuricemia and gout: males have a higher risk and incidence than females [42,43,44,45]. Estrogens seem to promote uric acid excretion and gout prevention [46,47]. Based on this effect, it can be inferred that since males are more likely to suffer from diseases related to purine metabolism, purines in the diet affect males more noticeably. A Japanese cohort study found a U-shaped relationship between blood uric acid levels and mortality in males, and the uric acid level at the lowest point of the curve (the third quintile) in that study was similar to the uric acid level in the top quintile of our study (327.14 vs. 329.95 μmol/L, respectively) [48]. This difference also suggests that the overall level of serum uric acid in the Chinese population is low, from which it can be inferred that purine intake is low in China. A similar conclusion was reached in a study in Korea [49].

One strength of our study is that it uses a large sample of nationally representative data, so the conclusions are relatively reliable. Moreover, the quantitative calculation of purine intake adopted in our study is more accurate than those in previous studies. However, our study also has some limitations. Since the *Chinese Food Composition Table* does not include the purine-content measurement values of all foods, we replaced the missing values with averages for the corresponding categories to calculate the specific purine intake, which may have introduced some errors. In addition, the effects of seasoning and cooking methods were not considered in the calculation of purine intake. Finally, our study only explored the relationship between purines and all-cause mortality, but it did not further explore specific causes of mortality, such as cardiovascular disease, so further research is needed to determine whether the deaths were caused by purine intake.

Our research reveals the dose–response relationship between purine intake and death, suggesting that a higher purine intake may be related to lower mortality, depending on the food source of the purine and sex. This finding is meaningful for the development of strategies for preventing related diseases.

## 5. Conclusions

Considering the relatively low purine intake levels in the Chinese population, a higher purine intake was related to lower mortality, and the inverse associations with mortality were mainly found in purines derived from plant foods and in males. We observed a U-shaped relationship between purine intake and mortality in males, but purine intake may not relate to mortality in females.

## Figures and Tables

**Table 1:** Baseline characteristics of participants by quintiles of purine intake (*n* = 17,755) ^a^.

	Quintiles of Purine Intake					*p* for Trend ^b^
	Quintile 1	Quintile 2	Quintile 3	Quintile 4	Quintile 5	
*n*	3551	3551	3551	3551	3551	
Mean purine intake, mg/day	168.75 ± 36.22	246.87 ± 17.80	308.71 ± 18.43	380.00 ± 24.57	541.39 ± 136.97	<0.001
Age, year	51.18 ± 17.68	49.37 ± 16.47	48.60 ± 15.84	48.83 ± 15.28	48.26 ± 15.19	<0.001
Male, %	42.6	47.0	46.4	48.8	51.8	<0.001
Urban residence, %	31.5	36.2	39.6	43.9	50.2	<0.001
Income, CNY/year	11,953.33 ± 16,156.92	14,319.05 ± 19,170.48	15,366.37 ± 20,335.71	18,131.56 ± 25,167.13	21,743.00 ± 33,291.38	<0.001
Education year, year	7.16 ± 4.37	8.00 ± 4.19	8.35 ± 4.14	8.77 ± 4.07	9.10 ± 4.02	<0.001
Energy intake, kcal/day	1915.71 ± 720.66	2011.52 ± 658.91	1988.82 ± 629.17	1970.03 ± 588.96	1872.56 ± 548.00	0.069
Carbohydrate intake, g/day	271.74 ± 115.78	284.61 ± 111.10	272.41 ± 105.19	253.47 ± 93.56	220.59 ± 83.02	<0.001
Protein intake, g/day	53.69 ± 24.04	59.42 ± 22.03	63.03 ± 20.97	68.19 ± 21.47	75.81 ± 24.59	<0.001
Fat intake, g/day	65.69 ± 40.16	68.15 ± 36.61	70.24 ± 34.02	74.32 ± 33.38	74.50 ± 32.04	<0.001
BMI, kg/m^2^	23.41 ± 4.29	23.27 ± 3.52	23.23 ± 3.72	23.28 ± 3.41	23.37 ± 3.59	0.819
Smoke, %	29.4	30.5	30.1	31.7	34.3	<0.001
Drink alcohol, %	31.0	33.5	32.2	34.4	37.8	<0.001
Physical activity, MET-H/day	21.71 ± 19.72	23.05 ± 19.61	23.35 ± 17.70	22.98 ± 16.69	23.27 ± 16.05	<0.001
Diabetes, %	2.1	2.1	2.1	2.2	2.6	0.155
Hypertension, %	9.6	9.2	8.4	10.1	11.3	0.008
Uric acid, μmol/L	287.73 ± 95.60	303.95 ± 100.50	310.18 ± 101.35	313.01 ± 111.99	327.95 ± 119.06	<0.001
Person-year	8.73 ± 2.96	8.67 ± 2.92	8.44 ± 3.02	8.16 ± 3.09	7.61 ± 3.20	<0.001
Death, %	6.6	4.8	3.9	3.7	2.3	<0.001

^a^ Data are means ± SDs for continuous variables and % for categorical variables. ^b^ *p* for trend values were analyzed by Jonckheere–Terpstra test for continuous variables or Cochran–Armitage trend test for categorical variables.

**Table 2:** HRs (95% CIs) of mortality according to the quintiles of purine intake ^a^.

	Quintiles of Purine Intake					*p* for Trend ^b^
	Quintile 1	Quintile 2	Quintile 3	Quintile 4	Quintile 5	
Total purine						
Median intake, mg/day	179.43	251.44	313.39	384.92	514.94	
Deaths, cases/total	235/3551	171/3551	137/3551	132/3551	83/3551	
Model 1 ^c^	1.00	0.81 (0.67–0.99)	0.74 (0.60–0.92)	0.73 (0.59–0.91)	0.49 (0.38–0.63)	<0.001
Model 2 ^d^	1.00	0.90 (0.74–1.10)	0.85 (0.68–1.05)	0.87 (0.70–1.08)	0.60 (0.47–0.78)	<0.001
Model 3 ^e^	1.00	0.90 (0.74–1.10)	0.84 (0.68–1.04)	0.86 (0.70–1.07)	0.60 (0.46–0.77)	<0.001
Model 3 + protein intake	1.00	0.94 (0.77–1.14)	0.91 (0.73–1.13)	0.98 (0.78–1.23)	0.74 (0.56–0.99)	0.144
Animal-derived purine						
Median intake, mg/day	23.66	78.46	131.93	192.41	305.52	
Deaths, cases/total	268/4472	153/3321	140/3321	119/3321	78/3320	
Model 1	1.00	0.86 (0.70–1.04)	0.87 (0.71–1.07)	0.78 (0.63–0.97)	0.53 (0.41–0.69)	<0.001
Model 2	1.00	1.02 (0.83–1.25)	1.03 (0.84–1.27)	1.00 (0.80–1.24)	0.72 (0.56–0.93)	0.067
Model 3	1.00	1.03 (0.84–1.25)	1.03 (0.84–1.27)	0.99 (0.80–1.24)	0.71 (0.55–0.92)	0.052
Model 3 + protein intake	1.00	1.06 (0.87–1.30)	1.11 (0.90–1.38)	1.12 (0.89–1.41)	0.92 (0.69–1.23)	0.778
Plant-derived purine						
Median intake, mg/day	112.9	149.98	178.06	212.2	280.53	
Deaths, cases/total	197/3552	152/3551	149/3550	148/3551	112/3551	
Model 1	1.00	0.88 (0.71–1.09)	0.87 (0.70–1.08)	0.87 (0.70–1.08)	0.67 (0.53–0.85)	0.003
Model 2	1.00	0.92 (0.74–1.14)	0.86 (0.69–1.07)	0.88 (0.71–1.10)	0.64 (0.51–0.81)	0.001
Model 3	1.00	0.91 (0.73–1.13)	0.86 (0.69–1.07)	0.89 (0.71–1.10)	0.64 (0.51–0.81)	0.001
Model 3 + protein intake	1.00	0.93 (0.75–1.15)	0.90 (0.72–1.12)	0.94 (0.75–1.17)	0.71 (0.56–0.90)	0.019

^a^ Cox proportional hazard models were used to calculate the hazard ratios (HRs) and 95% CIs for death. ^b^ *p* for trend values were analyzed by Cox proportional hazard models. ^c^ Model 1 was adjusted for age and gender. ^d^ Model 2 was additionally adjusted for residence, income, education year, smoking status, drinking status, physical activity, BMI, and energy intake. ^e^ Model 3 was further adjusted for hypertension and diabetes.

**Table 3:** HRs (95% CIs) of mortality according to the quintiles of purine intake in males ^a^.

	Quintiles of Purine Intake					*p* for Trend ^b^
	Quintile 1	Quintile 2	Quintile 3	Quintile 4	Quintile 5	
Total purine						
Median intake, mg/day	182.52	251.83	314.81	385.98	517.37	
Deaths, cases/total	129/1512	97/1670	74/1649	80/1733	46/1841	
Model 1 ^c^	1.00	0.73 (0.56–0.95)	0.65 (0.49–0.87)	0.67 (0.51–0.89)	0.40 (0.29–0.56)	<0.001
Model 2 ^d^	1.00	0.78 (0.60–1.02)	0.73 (0.55–0.98)	0.77 (0.58–1.02)	0.48 (0.34–0.68)	<0.001
Model 3 ^e^	1.00	0.78 (0.60–1.02)	0.73 (0.54–0.97)	0.77 (0.58–1.02)	0.47 (0.34–0.67)	<0.001
Model 3 + protein intake	1.00	0.83 (0.64–1.09)	0.81 (0.61–1.09)	0.94 (0.69–1.27)	0.67 (0.46–0.97)	0.127
Animal-derived purine						
Median intake, mg/day	8.65	83.28	136.66	195.74	311.35	
Deaths, cases/total	153/2032	84/1509	71/1545	69/1610	49/1709	
Model 1	1.00	0.81 (0.62–1.06)	0.72 (0.54–0.95)	0.72 (0.54–0.95)	0.51 (0.37–0.70)	<0.001
Model 2	1.00	0.97 (0.74–1.27)	0.84 (0.63–1.12)	0.91 (0.68–1.21)	0.67 (0.48–0.93)	0.024
Model 3	1.00	0.97 (0.74–1.27)	0.84 (0.63–1.12)	0.91 (0.68–1.21)	0.66 (0.48–0.92)	0.022
Model 3 + protein intake	1.00	1.02 (0.78–1.34)	0.94 (0.70–1.26)	1.10 (0.81–1.49)	0.99 (0.68–1.43)	0.876
Plant-derived purine						
Median intake, mg/day	107.24	148.35	185.21	219.1	290.85	
Deaths, cases/total	94/1423	88/1574	89/1731	90/1799	65/1878	
Model 1	1.00	0.96 (0.72–1.28)	0.89 (0.67–1.19)	0.90 (0.67–1.20)	0.63 (0.46–0.87)	0.007
Model 2	1.00	0.96 (0.71–1.28)	0.84 (0.62–1.12)	0.86 (0.64–1.16)	0.58 (0.42–0.80)	0.001
Model 3	1.00	0.95 (0.71–1.27)	0.84 (0.63–1.13)	0.87 (0.65–1.16)	0.58 (0.42–0.79)	0.001
Model 3 + protein intake	1.00	0.98 (0.73–1.31)	0.89 (0.66–1.20)	0.94 (0.70–1.27)	0.67 (0.48–0.92)	0.027

^a^ Cox proportional hazard models were used to calculate the hazard ratios (HRs) and 95% CIs for death. ^b^ *p* for trend values were analyzed by Cox proportional hazard models. ^c^ Model 1 was adjusted for age. ^d^ Model 2 was additionally adjusted for residence, income, education year, smoking status, drinking status, physical activity, BMI, and energy intake. ^e^ Model 3 was further adjusted for hypertension and diabetes.

**Table 4:** HRs (95% CIs) of mortality according to the quintiles of purine intake in females ^a^.

	Quintiles of Purine Intake					*p* for Trend ^b^
	Quintile 1	Quintile 2	Quintile 3	Quintile 4	Quintile 5	
Total purine						
Median intake, mg/day	176.76	251.16	312.09	384.04	512.74	
Deaths, cases/total	106/2039	74/1881	63/1902	52/1818	37/1710	
Model 1 ^c^	1.00	0.94 (0.70–1.26)	0.87 (0.63–1.19)	0.82 (0.59–1.14)	0.64 (0.44–0.94)	0.019
Model 2 ^d^	1.00	1.09 (0.81–1.47)	1.01 (0.74–1.39)	1.04 (0.74–1.46)	0.83 (0.56–1.21)	0.454
Model 3 ^e^	1.00	1.09 (0.81–1.47)	1.00 (0.73–1.38)	1.03 (0.74–1.45)	0.82 (0.56–1.20)	0.415
Model 3 + protein intake	1.00	1.10 (0.81–1.49)	1.03 (0.74–1.42)	1.07 (0.75–1.53)	0.88 (0.57–1.35)	0.773
Animal-derived purine						
Median intake, mg/day	1.12	68.86	115.99	170.03	277.6	
Deaths, cases/total	115/2440	69/1812	69/1776	50/1711	29/1611	
Model 1	1.00	0.92 (0.68–1.24)	1.09 (0.81–1.47)	0.88 (0.63–1.22)	0.57 (0.38–0.86)	0.030
Model 2	1.00	1.09 (0.81–1.48)	1.31 (0.97–1.78)	1.14 (0.81–1.60)	0.81 (0.53–1.23)	0.965
Model 3	1.00	1.10 (0.81–1.49)	1.32 (0.98–1.79)	1.12 (0.80–1.58)	0.80 (0.52–1.21)	0.879
Model 3 + protein intake	1.00	1.11 (0.82–1.51)	1.36 (1.00–1.85)	1.17 (0.82–1.65)	0.87 (0.55–1.37)	0.693
Plant-derived purine						
Median intake, mg/day	95.77	135.03	164.62	197.46	263.31	
Deaths, cases/total	103/2129	64/1977	60/1819	58/1752	47/1673	
Model 1	1.00	0.80 (0.59–1.10)	0.86 (0.62–1.18)	0.85 (0.62–1.18)	0.74 (0.53–1.05)	0.140
Model 2	1.00	0.88 (0.64–1.21)	0.91 (0.66–1.26)	0.95 (0.68–1.32)	0.77 (0.54–1.10)	0.257
Model 3	1.00	0.87 (0.64–1.20)	0.90 (0.65–1.25)	0.95 (0.68–1.32)	0.77 (0.54–1.10)	0.275
Model 3 + protein intake	1.00	0.88 (0.64–1.20)	0.91 (0.66–1.27)	0.96 (0.69–1.35)	0.80 (0.56–1.16)	0.392

^a^ Cox proportional hazard models were used to calculate the hazard ratios (HRs) and 95% CIs for death. ^b^ *p* for trend values were analyzed by cox proportional hazard models. ^c^ Model 1 was adjusted for age. ^d^ Model 2 was additionally adjusted for residence, income, education year, smoking status, drinking status, physical activity, BMI, and energy intake. ^e^ Model 3 was further adjusted for hypertension and diabetes.

## Data Availability

The data were obtained from CHNS. The original database is available at the website (http://www.cpc.unc.edu/projects/China) (accessed on 1 March 2022).

## Supplementary Materials

The following supporting information can be downloaded at: https://www.mdpi.com/article/10.3390/nu14091718/s1, Table S1: HRs (95% CIs) of mortality according to the quintiles of purine-rich food intake.
