# Supplementary material for: The Association between Dietary Purine Intake and Mortality: Evidence from the CHNS Cohort Study
Source: Nutrients. 2022 Apr 21;14(9):1718. doi: 10.3390/nu14091718 (PMC9102343; doi:10.3390/nu14091718)
Supplement: Supplementary file 1 [file nutrients-14-01718-s001.zip › nutrients-1642794-supplementary.pdf]

Table S1 HRs (95% CIs) of mortality according to the quintiles of purine-rich food intake[a]

	Quintiles of purine-rich food intake					*P* for trend[b]
	Quintile 1	Quintile 2	Quintile 3	Quintile 4	Quintile 5	
Red meat						
Median intake, g/day	50.00	89.17	116.11	150.00	207.50	
Deaths, cases/total	281/4851	156/3647	95/2812	116/3219	110/3226	
Model 1[c]	1.00	**0.71(0.58,0.86)**	**0.56(0.44,0.71)**	**0.64(0.52,0.80)**	**0.65(0.52,0.81)**	**<0.001**
Model 2[d]	1.00	**0.79(0.65,0.96)**	**0.62(0.49,0.78)**	**0.74(0.59,0.93)**	**0.73(0.58,0.91)**	**<0.001**
Model 3[e]	1.00	0.86(0.71,1.05)	**0.74(0.58,0.94)**	0.88(0.70,1.10)	0.86(0.68,1.09)	0.142
Poultry						
Median intake, g/day	50.00	85.00	100.00	150.00	250.00	
Deaths, cases/total	596/11857	18/535	68/2285	28/1293	48/1785	
Model 1	1.00	0.65(0.41,1.04)	**0.63(0.49,0.80)**	**0.50(0.34,0.74)**	**0.58(0.43,0.77)**	**<0.001**
Model 2	1.00	0.75(0.47,1.19)	**0.73(0.57,0.94)**	**0.59(0.40,0.87)**	**0.69(0.51,0.92)**	**<0.001**
Model 3	1.00	0.73(0.46,1.17)	0.78(0.60,1.01)	**0.63(0.43,0.93)**	0.76(0.56,1.03)	**0.003**
Seafood						
Median intake, g/day	60.00	100.00	150.00	200.00	291.67	
Deaths, cases/total	493/9627	62/2019	70/2093	76/1995	57/2021	
Model 1	1.00	**0.61(0.47,0.79)**	**0.64(0.50,0.83)**	**0.69(0.55,0.89)**	**0.52(0.40,0.69)**	**<0.001**
Model 2	1.00	**0.72(0.55,0.94)**	0.77(0.60,1.00)	0.83(0.65,1.06)	**0.63(0.48,0.84)**	**<0.001**
Model 3	1.00	0.78(0.60,1.03)	0.88(0.68,1.14)	0.97(0.76,1.25)	**0.74(0.56,0.98)**	0.065
Legumes						
Median intake, g/day	50.00	100.00	138.33	185.83	260.00	
Deaths, cases/total	329/6234	117/2781	114/2765	91/2816	107/2830	
Model 1	1.00	**0.72(0.58,0.89)**	**0.60(0.49,0.75)**	**0.50(0.40,0.64)**	**0.59(0.47,0.74)**	**<0.001**
Model 2	1.00	**0.77(0.62,0.95)**	**0.64(0.51,0.79)**	**0.54(0.43,0.69)**	**0.70(0.56,0.88)**	**<0.001**
Model 3	1.00	0.83(0.67,1.03)	**0.69(0.55,0.85)**	**0.58(0.46,0.73)**	**0.72(0.57,0.90)**	**<0.001**

Purine-rich vegetables and fungi						
Median intake, g/day	20.00	50.00	75.00	100.00	175.00	
Deaths, cases/total	658/12608	30/1330	17/1272	32/1350	21/1195	
Model 1	1.00	**0.46(0.32,0.67)**	**0.28(0.17,0.46)**	**0.48(0.34,0.69)**	**0.36(0.23,0.56)**	**<0.001**
Model 2	1.00	**0.61(0.42,0.88)**	**0.36(0.22,0.58)**	**0.59(0.41,0.85)**	**0.47(0.30,0.73)**	**<0.001**
Model 3	1.00	**0.61(0.42,0.88)**	**0.38(0.23,0.61)**	**0.66(0.46,0.94)**	**0.53(0.34,0.82)**	**<0.001**

[a] Cox proportional hazard models were used to calculate the hazard ratios (HRs) and 95% CIs for death

[b] P for trend values were analyzed by cox proportional hazard models

[c] Model 1 was adjusted for age and gender

[d] Model 2 additionally adjusted for residence, income, education year, smoking status, drinking status, physical activity, BMI, and energy intake

[e] Model 3 further adjusted hypertension, diabetes and 4 other types of purine-rich food intakes
